# Microstructure and Fatigue Behaviors of Dissimilar A6061/Galvannealed Steel Joints Fabricated by Friction Stir Spot Welding

**DOI:** 10.3390/ma14143877

**Published:** 2021-07-12

**Authors:** Koki Kumamoto, Tsuyoshi Kosaka, Tatsuya Kobayashi, Ikuo Shohji, Yuichiro Kamakoshi

**Affiliations:** 1 Graduate School of Science and Technology, Gunma University, Kiryu 3768515, Japan; t191b028@gunma-u.ac.jp (K.K.); t211b031@gunma-u.ac.jp (T.K.); kobayashi.t@gunma-u.ac.jp (T.K.); 2 Gunma Prefectural Industrial Technology Center, Maebashi 3792147, Japan; kamakoshi-yu@pref.gunma.lg.jp

**Keywords:** FSSW, dissimilar welding, tensile shear, tensile shear fatigue, A6061, galvanized steel, microstructure, intermetallic compounds

## Abstract

The microstructures, tensile shear properties, and tensile shear fatigue properties of dissimilar A6061/Galvannealed steel joints fabricated by friction stir spot welding (FSSW) were investigated. Fe_4_Al_13_ phases form as the intermetallic compound (IMC) layer at the joint interface between the A6061 matrix and the galvannealed layer consisting of FeZn_7_, Fe, and Zn. At the edge of the joint, the stirred layer in which the A6061 matrix and the galvannealed layer are stirred also forms. Moreover, the solidified part of the residual melt discharged from the joint area forms at the outer peripheries of the joint. In this study, FSSW was conducted for two total welding durations: 9 and 10 s. Although the thickness of the remaining A6061 sheet in the welded area decreased with an increase in the welding time, the effects of the total welding time on tensile shear and tensile shear fatigue properties were negligible. A fatigue fracture occurred in the A6061 matrix and at the joint interface at the high cycle fatigue region and the low cycle fatigue region, respectively. In the case of the interfacial fracture, the crack was generated in the solidified part of the residual melt or at the interface between the solidified part and the stirred layer.

## 1. Introduction

Making car bodies lightweight is expected to reduce carbon dioxide emissions as a method of slowing global warming [1]. Thus, multi-material technology that uses various materials for suitable parts in the car body has attracted attention [1,2,3]. Aluminum is a light metal, and its specific gravity is approximately one-third of iron. In addition, it has large specific strength, relatively good workability, and excellent corrosion resistance. Thus, using Al alloys has been expected to lighten car bodies. Since it is difficult to make the whole body using Al alloys [4], the realization of a hybrid construction between steel and Al alloys that maintains their respective advantages, has attracted attention. In previous studies, various methods of Al/steel welding have been investigated: ultrasonic spot welding for 6061 Al alloy and AISI 304 stainless steel [5], MIG-TIG double-side arc welding for 5052 Al alloy and steel [6], explosive welding for 1230 Al alloy and AISI 321 stainless steel [7], resistance spot welding (RSW) for 6022 Al alloy and steel [8] and for 5052 Al alloy and GA590 steel [9], magnetic pulse welding for 5052 Al alloy and steel [10], friction melt bonding for 6061 Al alloy and DP980 steel [11], friction stir welding (FSW) for 5052 Al alloy and DP steel [12], etc.

In particular, to combine steel and Al alloys in the car body, fastening with a rivet (self-piercing riveting (SPR)) [13,14], laser welding [15,16] and RSW have been extensively researched and developed. In the SPR method, dissimilar sheet materials are joined by mechanical interlock using rivets. Thus, the technology has spread as an alternative method to conventional spot welding for materials that are difficult to weld [17]. The SPR method has been applied to join Al alloy and steel, and dissimilar Al alloys in the automobile field [3]. Various process improvements have also been conducted to improve the quality of the SPR joint [13,18]. Moreover, the mechanical properties of the SPR joints with high-strength steel and an Al alloy, and the effect of sheet strength and sheet thickness on those properties were reported [14]. The SPR joint reportedly shows relatively high tensile shear strength [14] and excellent fatigue properties [3,14]. However, the SPR method has a disadvantage in that the weight and cost of rivets increase with an increase in the number of joints.

In contrast, although the RSW method has been used in the welding of steel for car bodies, it is not easy to apply it in a dissimilar bond due to differences in melting points, thermal conductivities, coefficients of thermal expansion, etc. between materials. Additionally, the formation of Fe–Al brittle intermetallic compounds (IMCs) in the joint interface exerts a bad effect on the reliability of the joint [9,19,20]. Since the laser welding system unit can be replaced with the RSW system unit, laser welding of Al alloys and steel has attracted attention and much research has been conducted [15,16].

Under such a background, friction stir spot welding (FSSW) is expected to be a new method to overcome the above-mentioned problems. The FSSW method is derived from the FSW method. The FSW method has been already applied to the welding of dissimilar Al alloys [21], dissimilar steel [22,23], and Al alloy and steel [12,24]. FSW is a reliable welding method and has advantages in that the method does not require additions such as rivets and, thus, in that the process cost can be reduced compared to that of SPR. In FSSW, the tool stirs the Al alloy and creates an active surface on the Al alloy, and thus, the Al alloy is joined to steel by hot joining with a welding force [25,26]. In FSSW, the heat generated in the welding interface is small compared to that of RSW, and thus, the growth of IMC formed in the welded interface can be restrained. For mechanical properties of FSSW joints, tensile shear load [25,27], cross tension load [25,27], and tensile strength of microscale specimens [26,28] have been investigated. However, there are a few reports of fatigue properties of FSSW joints for 5052 Al alloy and DP980-GA steel [3], 6061 Al alloy and SPCC steel [29], and 5052 Al alloy and SPC270C steel [30]. Therefore, the aim of this study is to investigate the fatigue properties of an FSSW joint. The microstructures, tensile shear properties, and fatigue properties of FSSW joints with the A6061 alloy and GA980 galvannealed steel were investigated.

## 2. Materials and Methods

**Figure 1:**
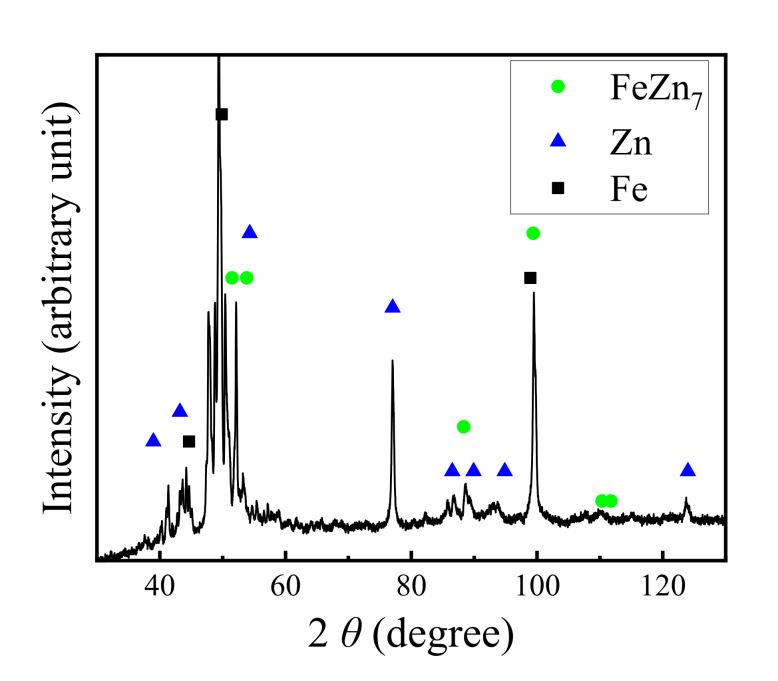
XRD analysis results for the surface of GA980.

An A6061 alloy sheet (Al–Mg–Si system) and a GA980 galvannealed steel sheet were prepared as an Al alloy and steel, respectively. The thicknesses of the A6061 sheet and the GA980 sheet were 1.6 and 1.4 mm, respectively. The thickness of the plating layer of the GA980 sheet was approximately 7 μm. Figure 1 shows the result of X-ray diffraction (XRD) analysis using a XRD system (SmartLab GK/2, Rigaku Corp., Tokyo, Japan) for the surface of the GA980 sheet. Co Kα X-ray was used in the measurement. FeZn_7_, Fe, and Zn were detected from the galvannealed layer. Thus, it was confirmed that the main reaction phase in the galvannealed layer of the GA980 sheet investigated in this study was FeZn_7_.

**Figure 2:**
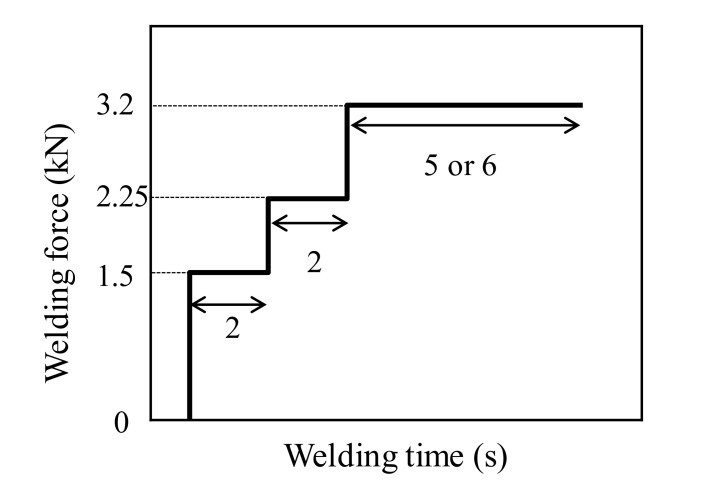
Relation between welding force and welding time in FSSW.

FSSW was conducted using a pin with a Φ2 mm tip diameter at 3000 rpm. This parameter was referenced from that used in a previous study that conducted FSSW of an A6061 alloy and hot dip galvanized steel [26,31]. Figure 2 shows the welding force profile during the welding process. In this study, the welding force was raised stepwise as shown in Figure 2. For this profile, the setting value was also referenced from a previous study [26]. Since the galvannealed layer is harder than the galvanized layer, a slightly higher welding force was set in this study compared with that applied to the galvanized layer [26]. From the feasibility study, in which both welding times of the first step and the second step shown in Figure 2 are 2 s, it was found that the tensile shear strength of the joint increases with an increase in the welding time of the third step and becomes the maximum at the total welding time of 10 s. Additionally, excess deformation of the A6061 alloy sheet was found when the total welding time was 12 s. Therefore, the total welding times were set to 9 and 10 s to investigate the mechanical properties of sound FSSW joints.

**Figure 3:**
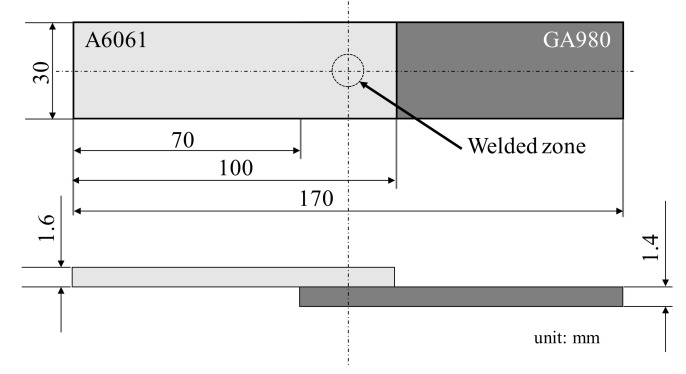
Shape and dimensions of the tensile shear specimen.

A tensile shear specimen shown in Figure 3 was fabricated by FSSW. To investigate the microstructure of the cross section of the FSSW joint, the joint was polished with #800–#4000 waterproof abrasive papers and was subsequently polished using 1 μm alumina powder suspension. Microstructural observation was conducted for the FSSW joint using a laser microscope (VK-X150, KEYENCE Corp., Osaka, Japan) and an electron probe X-ray microanalyzer (EPMA) (EPMA-1610, Shimadzu Corp., Kyoto, Japan). In addition, the A6061 alloy in the joint was etched with a sodium hydroxide aqueous solution (200 g/L) to investigate the fusion area. The solution temperature was 70 °C, and the etching time was from 10 to 15 min.

The tensile shear test was conducted using a universal testing machine (5567, Instron Japan Co., Ltd., Kanagawa, Japan). The cross head speed and test temperature were 5 mm/min and room temperature (R.T.), respectively. Two specimens were tested in each welding condition. After the tensile shear test, the fracture mode was analyzed using the laser microscope and the EPMA. Moreover, XRD analysis was conducted on the fracture surface of the specimen, which showed an interfacial fracture to investigate the formation phases in the joint interface.

The tensile shear fatigue test was conducted by load control using a sine wave with a fatigue testing machine (EHF-E, Shimadzu Corp., Kyoto, Japan). The frequency, stress ratio, and test temperature were 10 Hz, 0.1, and R.T., respectively. The test was conducted at load amplitudes of 1.5, 2.7, 4.0, and 5.0 kN to obtain a fatigue life at the 10^6^ level. After the tensile shear fatigue test, the fracture mode was analyzed using the laser microscope and the EPMA.

## 3. Results and Discussion

### 3.1. Microstructures of Joints

**Figure 4:**
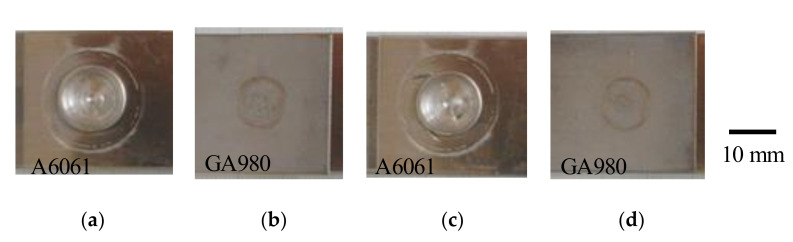
General views of the specimens of FSSW joints. (**a**) Welding time: 9 s, A6061 side; (**b**) welding time: 9 s, GA980 side; (**c**) welding time: 10 s, A6061 side and (**d**) welding time: 10 s, GA980 side.

**Figure 5:**
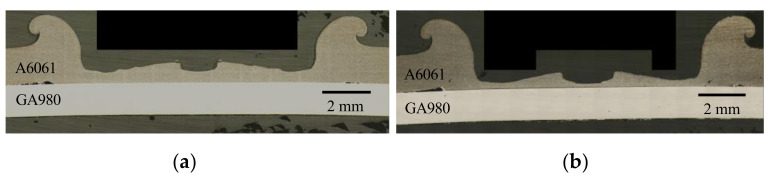
Optical microscope images of cross sections of FSSW joints. (**a**) Welding time: 9 and (**b**) welding time: 10 s.

**Figure 6:**
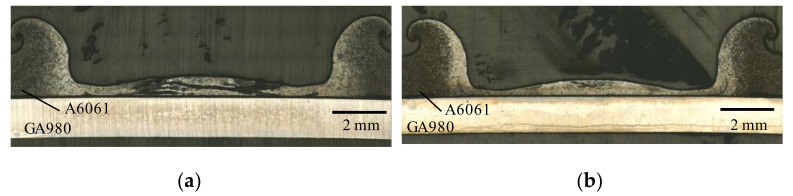
Optical microscope images of cross sections of the FSSW joints shown in Figure 5 after etching treatment. (**a**) Welding time: 9 s and (**b**) welding time: 10 s.

Figure 4 shows general views of FSSW joints. The views of (a), (b), (c) and (d) in Figure 4 were obtained from the same specimens. A large deformation was observed on the surface of the A6061 sheet at both welding times. On the surfaces of the GA980 sheets, a concave mark was not observed although a compression mark was observed. Cross sections of the FSSW joints are shown in Figure 5. The decrease in the thickness of the A6061 sheet was found to be remarkable and to increase with an increase in the welding time. The thickness of the A6061 sheet became less than half of the original thickness. Figure 6 shows the etched images with the sodium hydroxide aqueous solution for the cross sections shown in Figure 5. The bright areas in the A6061 alloy correspond to fusion areas. The contact area with a FSSW tool was found to have melted. Although the fusion zone in the joint welded at a welding time of 10 s is slightly larger than that welded at a welding time of 9 s, the difference is small.

**Figure 7:**
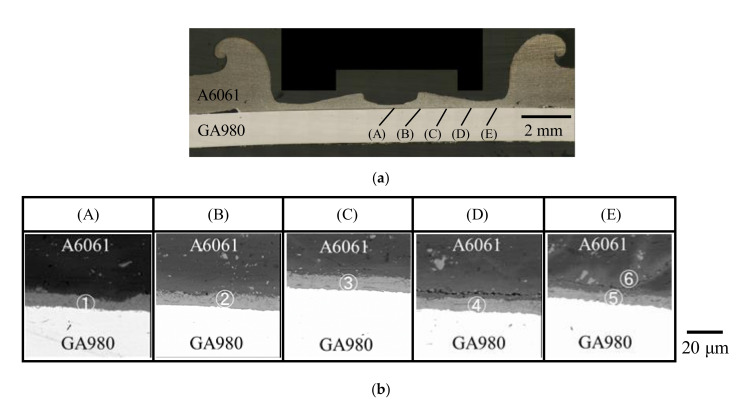
Microstructural observation result for the cross section of a joint using the laser microscope and the EPMA for the A6061/GA980 joint welded at welding time of 10 s. (**a**) Optical microscope image and analysis areas for EPMA analysis and (**b**) BSE images of the A6061/GA980 joint interface.

For the interfacial reaction layer formed in the FSSW joint shown in Figure 5b, an analysis using the EPMA was conducted. The results are shown in Figure 7 and Table 1. The backscattered electron (BSE) images were observed at the five areas from the center towards the edge with a 1 mm pitch, as shown in Figure 7b. The bright gray phases and the dark gray phases were observed in the interfacial reaction layer. On the basis of the result of the mapping analysis using the EPMA, the dark gray phase was inferred to be the condensed phase of Zn. In addition, the content of Zn in the reaction layer increased from the center to the edge. From the result of the quantitative analysis shown in Table 1, the bright gray phase in the interfacial reaction layer was inferred to be Fe_4_Al_13_ [32]. The formation of the similar Fe–Al IMC layer was reported in the dissimilar aluminum/steel joint fabricated via a refilled FSSW [33]. The formation of Fe_4_Al_13_ has also been reported in a previous study in which transmission electron microscope observations were conducted for a dissimilar aluminum/steel joint using the variable polarity cold metal transfer technique [34]. A similar microstructure was observed in the joint welded at welding time of 9 s.

**Figure 8:**
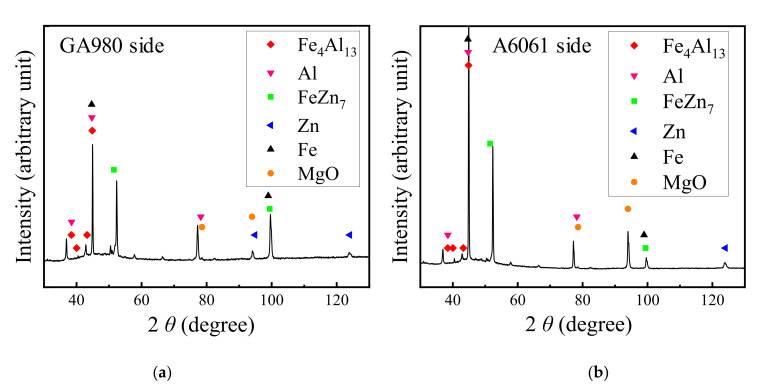
XRD analysis results for fracture surfaces ruptured by interfacial fracture in the tensile shear test with a joint welded at a welding time of 10 s. (**a**) Fracture surface of the GA980 sheet side and (**b**) fracture surface of the A6061 sheet side.

**Figure 9:**
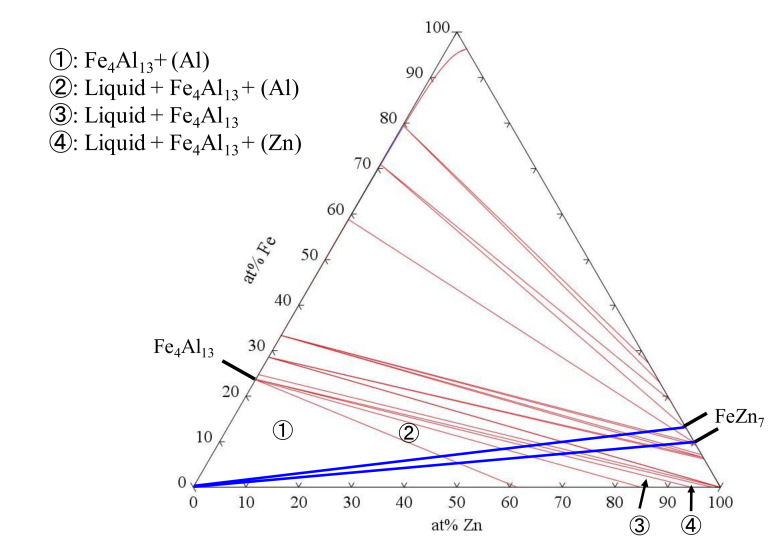
Isothermal section of the phase diagram of Fe–Zn–Al at 400 °C calculated by Thermo-Calc 2021a.

To identify the IMC formed at the interfacial reaction layer, an XRD analysis was conducted to the surfaces ruptured by interfacial fracture during the tensile shear test described later. The results of the XRD analysis are shown in Figure 8. Fe_4_Al_13_ was detected in both fracture surfaces, and thus, it was confirmed that Fe_4_Al_13_ phases form at the joint interface. In the FSSW process, the oxide film of A6061 is destroyed due to plastic flow of the softened A6061 matrix and due to heating by frictional heat, and thus, the active nascent surface is exposed. According to the Fe–Zn phase diagram [35] and the Al–Zn phase diagram [36], the transformation temperature of FeZn_7_ that is the main component of the plating layer of galvannealed steel is from 530 to 665 °C and the eutectic temperature of Al-Zn is 381 °C. Plastic flow at the stirred area has been reported to occur at approximately 400 °C in the friction stir welding of the A1100 sheet and the SS400 mild steel sheet [36]. Since the plastic flow of the A6061 matrix occurs in this study, the temperature in the stirred area seems to increase to at least around 400 °C. The isothermal section of the phase diagram of Fe–Zn–Al at 400 °C, calculated by Thermo-Calc 2021a, is shown in Figure 9. Considering that the temperature of the A6061/GA980 interface becomes more than 400 °C by friction heat, the plating layer of galvannealed steel contacts the active nascent surface of A6061 and thus Al reacts with FeZn_7_. From Figure 9, although Fe_4_Al_13_ forms on the Al-rich side, liquid (L)+Fe_4_Al_13_+(Al), L+Fe_4_Al_13_, and L+Fe_4_Al_13_+(Zn) form on the FeZn_7_-rich side depending on the ratio of Al and FeZn_7_, and thus, the L phase appears at the welding interface. Once such an L phase is removed from the welding interface by pressure and rotation of the pin and shoulder of the FSSW tool, both active nascent surfaces of A6061 and GA980 make contact and are bonded in solid state, and thus, the reaction layer including Fe_4_Al_13_ forms at the joint interface.

At the edge that corresponds to (E) shown in Figure 7a, the formation of another layer was observed between A6061 and the interfacial reaction layer. In another layer that corresponds to the area ➅ shown in Figure 7b, a few types of phases are also present. The identification of each phase was difficult because the size of each phase was too small to conduct the EPMA quantitative analysis. From the result of the quantitative analysis shown in Table 1, the area ➅ was found to include 11.2 at% Zn although the main component is Al. This means that the area ➅ is the stirred layer that consists of A6061 and the plating layer of the galvannealed steel. In addition, microcracks were observed in the stirred layer at the edge of the joint, as shown in areas (D) and (E) in Figure 7b. The liquation cracking reportedly generates in the FSSW joint of A6061 and galvanized sheet steel during the welding process [31]. In the joint, liquid phase generates by melting the Zn layer when the welding temperature increases. Thus, liquation cracking is generated by shear force caused by stirring or a restraining force added during solidification. In this study, although the galvannealed layer of the GA980 sheet is mainly FeZn_7_, the eutectic reaction with Al in A6061 and Zn in the galvannealed layer occurs and, thus, the eutectic melt is generated. Thus, liquation cracking is possibly generated in this study. Therefore, the microcracks observed in the edge area where the melt is discharged seem to be generated by liquation cracking. A similar microstructure was also observed in the joint welded at the welding time of 9 s.

### 3.2. Tensile Shear Properties

**Figure 10:**
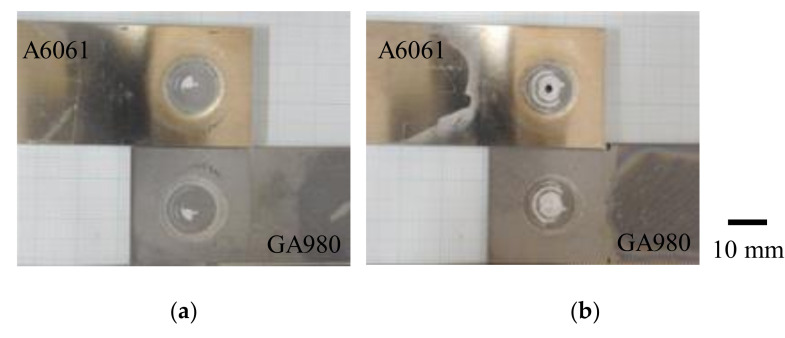
Examples of typical fracture surfaces by tensile shear test. (**a**) A6061/GA980 interfacial fracture (welding time: 9 s, specimen No.: No. 2) and (**b**) plug destruction (welding time: 10 s, specimen No.: No. 2).

The results of the tensile shear test are shown in Table 2. The effect of the welding time on tensile shear force is negligible although the thicknesses of the remainder of the A6061 sheet in the welded area are different depending on the welding time (refer to Figure 5). In addition, both plug fracture and interfacial fracture were observed in the specimens regardless of welding time. Figure 10 shows the typical fracture modes observed in this study. In the feasibility study, obtaining a sound joint in a shorter welding time was difficult. This means that the interfacial fracture mainly occurs when the welding time is less than 9 s. In contrast, from Figure 5, one can see that excess deformation of the A6061 sheet occurs when the welding time is more than 10 s. Thus, a fracture in the thin area of the A6061 sheet easily occurs. Therefore, the conditions of welding times of 9 and 10 s seem to be marginal conditions in which a sound joint is obtained. As a result, both interfacial fracture and plug destruction were observed in the cases with welding times of 9 and 10 s. In the previous study, the tensile forces of FSSW joints were reported to be approximately 5 kN for the joint with AA6063/galvanized steel [25] and 5–5.4 kN for those joints with A6061/galvanized steel [31]. The tensile force obtained in this study is slightly higher than those values. Therefore, a sound FSSW joint with A6061 and galvannealed steel was reported to have almost the same strength compared to that with A6061 and galvanized steel.

**Figure 11:**
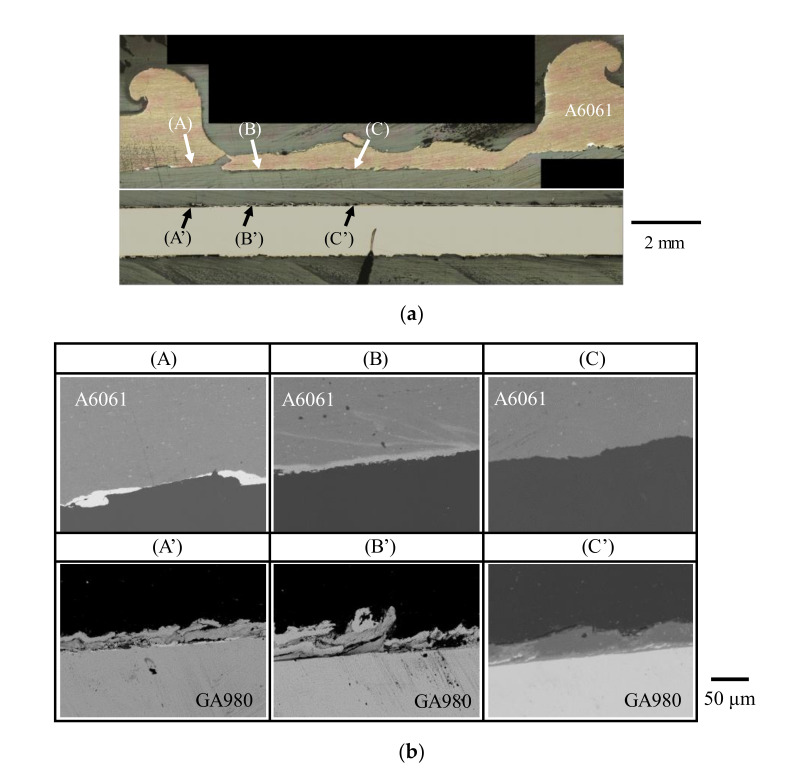
Cross-sectional image of the specimen ruptured by interfacial fracture in the tensile shear test (welding time: 10 s, specimen No.: No. 1). (**a**) General view from optical microscope imaging and the analyzed area using EPMA and (**b**) BSE images of the areas shown in Figure 11a observed with EPMA.

To investigate the details of the fracture mode in the interfacial fracture, a cross sectional observation was conducted for the joint ruptured by interfacial fracture. Figure 11 shows the results of the optical microscope image and BSE images of the cross section. The areas (C) and (C′) are the A6061 side and the GA980 side at the center of the joint, respectively. Only the matrix of A6061 was observed at area (C), and the stirred layer and Fe_4_Al_13_ were observed at area (C′). The areas (B) and (B′) are the A6061 side and the GA980 side at the edge of the joint, respectively. Only the stirred layer was observed at area (B), and the stirred layer and Fe_4_Al_13_ were observed at area (B′). In addition, area (B’) was rolled up similar to a hangnail. The area was deformed by the tensile shear test. The areas (A) and (A′) correspond to the outer peripheries of the joint. In area (A), white BSE phases are observed. Those phases are solidified parts of the residual melt that is discharged. In area (A′) as well as area (B′), the stirred layer and Fe_4_Al_13_ were observed. From these results, the crack was confirmed to be generated in the solidified part of the residual melt or at the interface between the solidified part of the residual melt and Fe_4_Al_13_ and mainly progresses at the interface between the stirred layer and Fe_4_Al_13_. Additionally, the crack passed the interface between the stirred layer and the A6061 matrix at the center of the joint. Moreover, a rupture of the A6061 matrix was also observed at the edge between areas (A) and (B).

### 3.3. Tensile Shear Fatigue Properties 

**Figure 12:**
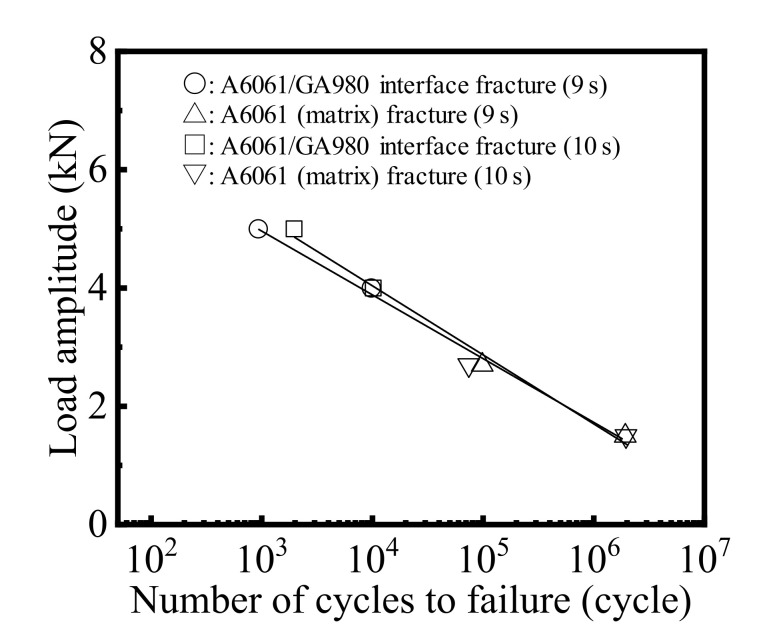
Results of the tensile shear fatigue test.

**Figure 13:**
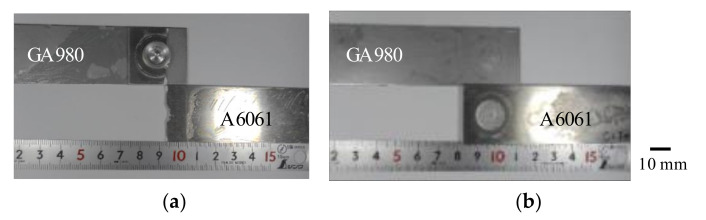
Examples of general views of fractured joints after the tensile shear fatigue test. (**a**) A6061 matrix fracture (welding time: 9 s, load amplitude: 2.7 kN, number of cycles to failure: 98,303 cycles) and (**b**) interfacial fracture (welding time: 10 s, load amplitude: 4.0 kN, number of cycles to failure: 10,165 cycles).

The load amplitude and the number of cycles to failure (*L*-*N*) diagram in the tensile shear fatigue test are shown in Figure 12. The effect of the welding time on the *L*-*N* diagram is negligible. When the number of cycles to failure is more than around 10^5^ cycles, fatigue fracture occurred in the A6061 matrix regardless of the welding time. Figure 13a shows an example of the general view of the joint ruptured by an A6061 matrix fracture. It was found that the crack progressed in the vertical direction for the tensile direction. A previous study on an A5052/SPC270C FSSW joint reported that the crack progressed in the A5052 matrix in approximately the vertical direction for the tensile direction and that a fracture occurred in the tensile shear fatigue test [30]. This is because that shear stress becomes the maximum along the neighborhood of the weld in the FSSW joint and the shear stress becomes relatively high compared with the stress over the real weld area with a decrease in the test load. As a result, the crack progressed in the A5052 matrix in the vertical direction for the tensile direction [30]. In this study, as shown in Figure 5, the thickness of the A6061 sheet became less than half of the original thickness at the edge of the joint. Thus, the withstanding load of the A6061 decreased at the edge of the joint. From the above-mentioned reason, it seems that the A6061 matrix fracture occurs easily at the high cycle fatigue region.

**Figure 14:**
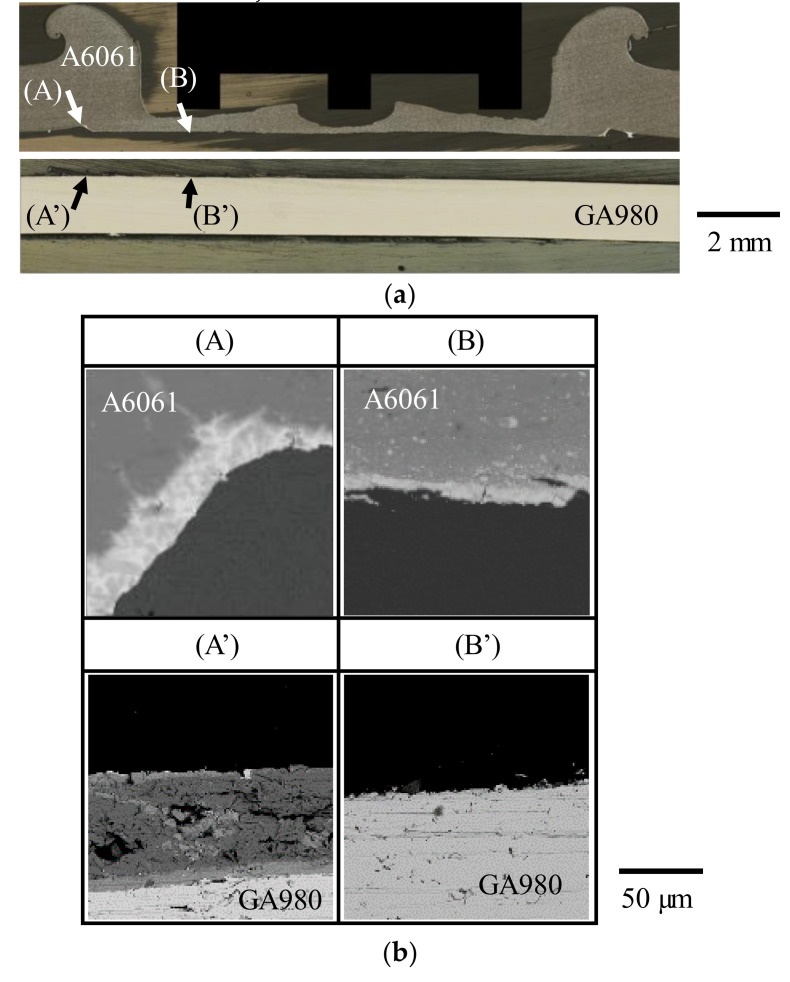
Cross-sectional image of the specimen ruptured by interfacial fracture in the tensile shear fatigue test (welding time: 10 s, load amplitude: 4.0 kN, number of cycles to failure: 10,165 cycles). (**a**) General view by optical microscope imaging and analyzed area by EPMA and (**b**) BSE images of the areas shown in Figure 14a observed with EPMA.

In contrast, when the number of cycles to failure is less than 10^5^ cycles, interfacial fracture occurred regardless of the welding time. A similar tendency was observed in the A5052/SPC270C FSSW joint [30]. An example of the general view of the joint ruptured by interfacial fracture is shown in Figure 13b. To investigate the detail of the fracture mode in the interfacial fracture using the tensile shear fatigue test, the cross section of the joint ruptured by interfacial fracture was observed. Figure 14 shows the result of the optical microscope image and BSE images of the cross section. The areas (A) and (A′) correspond to the outer peripheries of the ruptured joint on the A6061 and the GA980 sides, respectively. The solidified part of the residual melt was observed in area (A) and the stirred layer was observed in area (A′). The areas (B) and (B′) are the edges of the ruptured joint on the A6061 side and the GA980 sides, respectively. The stirred layer was observed in area (B), and a thin IMC layer was observed in area (B′). From those results, it seems that the crack was generated in the solidified part of the residual melt or at the interface between the solidified part and the stirred layer and progressed in the stirred layer and the interface between the stirred layer and the IMC layer, and thus, the joint finally fractured at the joint interface.

Similar interfacial fracture damage was also observed in the joint welded at the welding time of 9 s. Therefore, the crack resistance characteristics of the stirred layer seems to be one of main parameters in improving the tensile shear fatigue properties of an A6061/GA980 FSSW joint.

Table 3 shows a comparison of the fatigue strength at 10^5^ cycles of dissimilar FSSW joints with Al alloy and steel. Although the fatigue strength depends on the types and thicknesses of the dissimilar materials and the conditions of the fatigue test, the fatigue strength obtained in this study was confirmed to be superior or equal to those investigated in the previous studies.

## 4. Conclusions

In this study, a sound dissimilar FSSW joint with an A6061 alloy sheet and a GA980 galvannealed steel sheet was fabricated for total welding times of 9 and 10 s using a stepwise welding force profile. The microstructures, tensile shear properties and tensile shear fatigue properties of the FSSW joints were investigated. The results obtained are summarized as follows:

(1) Fe_4_Al_13_ phases form as the IMC layer at the joint interface between the A6061 matrix and the galvannealed layer of GA980 consisting of FeZn_7_, Fe and Zn. At the edge of the joint, the stirred layer, which consists of the A6061 matrix and the galvannealed layer, forms between the A6061 matrix and the IMC layer. Moreover, the solidification of the residual melt discharged from the joint area occurs at the outer peripheries of the joint.
(2) An analogous tensile shear force was obtained regardless of the welding time, although the thickness of the remainder of the A6061 sheet in the welded area decreased with an increase in the welding time. In the tensile shear test, the crack generated in the solidified part of the residual melt or at the interface between the solidified part of the residual melt and Fe_4_Al_13_ and mainly progressed at the interface between the stirred layer and Fe_4_Al_13_.
(3) Analogous fatigue properties were obtained regardless of the welding time. At the high cycle fatigue region in which the number of cycles to failure is more than around 10^5^ cycles, fatigue fracture occurred in the A6061 matrix. In contrast, at the low cycle fatigue region in which the number of cycles to failure is less than 10^5^ cycles, interfacial fracture occurred. In the case of the interfacial fracture, the crack is generated in the solidified part of the residual melt or at the interface between the solidified part and the stirred layer and progresses in the stirred layer and the interface between the stirred layer and the IMC layer.
(4) For the fatigue strength of 10^5^ cycles, the dissimilar FSSW joints investigated in this study are superior or equal to those of the dissimilar FSSW joints with Al alloy and steel investigated in previous studies.

## Figures and Tables

**Table 1:** Quantitative analysis results for the analysis points shown in Figure 7b.

Analysis Point	Al	Fe	Mg	Si	Zn (at%)	Inferred Phase
➀	75.0	22.6	0.3	0.4	1.7	Fe_4_Al_13_
➁	77.1	20.3	0.7	0.2	1.7	Fe_4_Al_13_
➂	76.2	21.5	0.1	0.5	1.8	Fe_4_Al_13_
➃	79.4	16.8	0.2	0.6	3.1	Fe_4_Al_13_
➄	76.4	17.8	1.1	0.8	3.9	Fe_4_Al_13_
➅	87.2	0.9	0.5	0.2	11.2	A6061 and GA plating

**Table 2:** Results of the tensile shear test.

Welding Time (s)	Specimen No.	Tensile Shear Force (kN)	Fracture Mode
9	No. 1	5.73	Plug destruction
	No. 2	5.52	A6061/GA980 interfacial fracture
10	No. 1	5.56	A6061/GA980 interfacial fracture
	No. 2	5.47	Plug destruction

**Table 3:** Comparison of the fatigue strength of dissimilar FSSW joints with Al alloy and steel.

Al Alloy		Steel		Fatigue Test Parameter			Fatigue Strength at 10^5^ Cycles	Reference
Type	Thickness (mm)	Type	Thickness (mm)	Frequency (Hz)	Stress Ratio	Wave		
A6061	1.6	GA980	1.4	10	0.1	sine	≈3 kN	this study
A5052	1.0	SPC270C	1.0	20	0.05	sine	≈1 kN	[30]
A5052	2.0	980DP-GA	1.4	10	0.05	sine	≈2 kN	[3]
A6061	2.0	SPCC	2.0	10	0.1	sine	2–2.5 kN	[29]
A6082	2.0	DP600	1.0	20	0.1	sine	≈3 kN	[37]

## Data Availability

The data are contained within this article.

## References

[1] Hirata Y. (2017). Welding and joining technology in government project of research and development on innovative structural materials operated by NEDO and ISMA. J. JWS.

[2] Martinsen K., Hu S.J., Carlson B.E. (2015). Joining of dissimilar materials. CIRP Ann. Manuf. Technol..

[3] Hirata Y. (2020). Comparison of joint performances by various dissimilar joining processes–research and development on welding and joining technology in government project operated by NEDO and ISMA (2). J. JWS.

[4] Watanabe T., Doi Y., Yanagisawa A., Konuma S. (2005). Resistance spot welding of mild steel to Al-Mg alloy. Q. J. Jpn. Weld. Soc..

[5] Mirza F.A., Macwan A., Bhole S.D., Chen D.L., Chen X.-G. (2016). Effect of welding energy on microstructure and strength of ultrasonic spot welded dissimilar joints of aluminum to steel sheets. Mater. Sci. Eng. A.

[6] Ye Z., Huang J., Cheng Z., Gao W., Zhang Y., Chen S., Yang J. (2017). Combined effects of MIG and TIG arcs on weld appearance and interface properties in Al/steel double-sided butt welding-brazing. J. Mater. Process. Technol..

[7] Shiran M.K.G., Khalaj G., Pouraliakbar H., Jandaghi M., Bakhtiari H., Shirazi M. (2017). Effects of heat treatment on the intermetallic compounds and mechanical properties of the stainless steel 321–aluminum 1230 explosive-welding interface. Int. J. Miner. Metall. Mater..

[8] Rao H.M., Kang J., Shi L., Sigler D.R., Carlson B.E. (2018). Effect of specimen configuration on fatigue properties of dissimilar aluminum to steel resistance spot welds. Int. J. Fatigue.

[9] Kumamoto K., Shohji I., Kobayashi T., Iyota M. (2021). Effect of microstructure on joint strength of Fe/Al resistance spot welding for multi-material components. Mater. Sci. Forum.

[10] Geng H., Sun L., Li G., Cui J., Huang L., Xu Z. (2019). Fatigue fracture properties of magnetic pulse welded dissimilar Al-Fe lap joints. Int. J. Fatigue.

[11] Sapanathan T., Jimenez-Mena N., Sabirov I., Monclús M.A., Molina-Aldareguía J.M., Xia P., Zhao L., Simar A. (2019). A new physical simulation tool to predict the interface of dissimilar aluminum to steel welds performed by friction melt bonding. J. Mater. Sci. Technol..

[12] Anaman S.Y., Cho H.-H., Das H., Lee J.-S., Hong S.-T. (2019). Microstructure and mechanical/electrochemical properties of friction stir butt welded joint of dissimilar aluminum and steel alloys. Mater. Charact..

[13] Haque R. (2018). Quality of self-piercing riveting (SPR) joints from cross-sectional perspective: A review. Arch. Civ. Mech. Eng..

[14] Zhang C.U., Gou R.B., Yu M., Zhang Y.J., Qiao Y.H., Fang S.P. (2017). Mechanical and fatigue properties of self-piercing riveted joints in high-strength steel and aluminium alloy. J. Iron Steel Res. Int..

[15] Xia H., Li L., Ma N., Tan C., Gong J. (2020). Influence of energy ratio on dual–spot laser welded–brazed Al/steel butt joint. J. Mater. Process. Technol..

[16] Qianqian G., Jiangqi L., Ping Y., Shunchao J., Wenhao H., Jianxi Z. (2019). Effect of steel to aluminum laser welding parameters on mechanical properties of weld beads. Opt. Laser Technol..

[17] He X., Pearson I., Young K. (2008). Self-pierce riveting for sheet materials: State of the art. J. Mater. Process. Technol..

[18] Zhang H. (2020). Influence of riveting sequence/direction on distortion of steel and aluminum sheets. J. Manuf. Process..

[19] Chen J., Feng Z., Wang H.P., Carlson B.E., Brown T., Sigler D. (2018). Multi-scale mechanical modeling of Al-steel resistance spot welds. Mater. Sci. Eng. A.

[20] Ogura T., Hirose A. (2016). Microstructural control of interface and mechanical properties in dissimilar metal joining between aluminum alloy and steel. J. Jpn. Inst. Light Met..

[21] Jandaghi M.R., Pouraliakbar H., Saboori A., Hong S.I. (2021). Comparative insight into the interfacial phase evolutions during solution treatment of dissimilar friction stir welded AA2198-AA7475 and AA2198-AA6013 aluminum sheets. Materials.

[22] El-Batahgy A.M., Miura T., Ueji R., Fujii H. (2016). Investigation into feasibility of FSW process for welding 1600 MP aquenched and tempered steel. Mater. Sci. Eng. A.

[23] Mira-Aguiar T., Verdera D., Leitão C., Rodrigues D.M. (2016). Tool assisted friction welding: A FSW related technique for the linear lap welding of very thin steel plates. J. Mater. Process. Technol..

[24] Kar A., Vicharapu B., Morisada Y., Fujii H. (2020). Elucidation of interfacial microstructure and properties in friction stir lap welding of aluminium alloy and mild steel. Mater. Charact..

[25] Piccini J.M., Svoboda H.G. (2015). Effect of pin length on friction stir spot welding (FSSW) of dissimilar aluminum-steel joints. Procedia Mater. Sci..

[26] Matsuda T., Owada K., Numata A., Shoji H., Sano T., Ohata M., Hirose A. (2020). Influence of interfacial structure on the fracture behavior of friction stir spot welded dissimilar joints. Mater. Sci. Eng. A.

[27] Dong H., Chen S., Song Y., Guo X., Zhang X., Sun Z. (2016). Refilled Friction Stir Spot Welding of Aluminum Alloy to Galvanized Steel Sheets. Mater. Des..

[28] Matsuda T., Sano T., Munekane M., Ohata M., Hirose A. (2020). Microscale tensile test of galvannealed steel/aluminum dissimilar joint for estimation of intrinsic interfacial strength. Scr. Mater..

[29] Uematsu Y., Tokaji K., Tozaki Y., Nakashima Y. (2010). Fatigue behaviour of dissimilar friction stir spot weld between A6061 and SPCC welded by a scrolled groove shoulder tool. Procedia Eng..

[30] Okane M., Shimizu T., Yasui T., Fukumoto M. (2018). Mechanistic factors influencing fatigue behavior of the A5052/SPC270C joints by friction stir spot welding. J. Light Met. Weld..

[31] Yamamoto M., Ogura T., Ohashi R., Fujimoto M., Hirose A. (2013). Effects of interfacial microstructures on joint strength in friction stir spot welded 6061 aluminum alloy/zinc coated steel joints. J. Light Met. Weld..

[32] Tobita K., Sato N., Kitahara K., Takagiwa Y., Kimura K. (2016). Effect of anomalous crystal structure of iron aluminides Fe_2_Al_5_ and Fe_4_Al_13_: Low phonon thermal conductivity and potentiality as thermoelectric materials. Mater. Trans..

[33] Li P., Chen S., Dong H., Ji H., Li Y., Guo X., Yang G., Zhang X., Han X. (2020). Interfacial microstructure and mechanical properties of dissimilar aluminum/steel joint fabricated via refilled friction stir spot welding. J. Manuf. Process..

[34] Xu P., Hua X., Shen C., Mou G., Li F. (2021). Formation of Fe_5_Si_3_ precipitate in the Fe_2_Al_5_ intermetallic layer of the Al/steel dissimilar arc welding joint: A transmission electron microscopy (TEM) study. Mater. Charact..

[35] Massalaki T.B., Okamoto H., Subramanian P.R., Kacprzak L. (2001). Binary Alloy Phase Diagrams.

[36] Massalaki T.B., Okamoto H., Subramanian P.R., Kacprzak L. (2001). Binary Alloy Phase Diagrams.

[37] Zhang Z., Yu Y., Zhao H., Tong H. (2020). Effect of loading methods on the fatigue properties of dissimilar Al/steel keyhole-free FSSW joints. Materials.

